# Winter Epibiotic Community of the Red King Crab *Paralithodes camtschaticus* in Sayda Bay (Barents Sea)

**DOI:** 10.3390/ani14010100

**Published:** 2023-12-27

**Authors:** Alexander G. Dvoretsky, Vladimir G. Dvoretsky

**Affiliations:** Murmansk Marine Biological Institute of the Russian Academy of Sciences (MMBI RAS), 183010 Murmansk, Russia

**Keywords:** *Paralithodes camtschaticus*, *Balanus crenatus*, *Ischyrocerus commensalis*, fouling community, fjord, Barents Sea, infestation indices, seasonal changes

## Abstract

**Simple Summary:**

Information on epibionts and symbionts of the introduced Barents Sea red king crab during the winter period is currently lacking. To address this gap, we examined the species composition and infestation indices of epibionts on this host in the northern part of Kola Bay in November. Our findings indicate a higher infestation rate of barnacles and amphipods during the winter season compared to the summer. In contrast, infestation rates of other species remained low during winter, reflecting seasonal fluctuations in both the size distribution of red king crabs and prevailing temperature conditions. Our research provides novel data on the growth patterns of commonly associated organisms, which could prove invaluable in determining the age of red king crabs in the Barents Sea.

**Abstract:**

The species composition of epibiotic communities on red king crab was investigated in Sayda Bay, Russia, during November of 2015 and 2016. The community consisted of 12 species in total. Among epibionts, the barnacle *Balanus crenatus* was most prevalent (67.0%), while the amphipod *Ischyrocerus commensalis* was the most frequent symbiont (77.3%). Infestation levels in May–June 2005 and September 2004 were higher, as a larger proportion of small crabs without fouling species were present during those seasons. The lower infestation intensities recorded for other common associated organisms during winter can be attributed to their increased mortality due to unfavorable temperature conditions. The localization of epibionts and symbionts were related to larval settlement patterns of attached species and feeding behavior of mobile species. Monthly growth increments for *I. commensalis* and *B. crenatus* were estimated at 2 mm in body length and 1.35 mm in basal diameter, respectively. Size-at-age data for epibionts can aid in the age determination of large male crabs that may skip an annual molt.

## 1. Introduction

The red king crab, *Paralithodes camtschaticus* (Tilesius, 1815), is a highly valued large decapod species. It was introduced into the Barents Sea due to its commercial significance, where it has formed a self-sustaining population [1]. Currently, the red king crab supports a regional fishery characterized by relatively high harvests of approximately 11,000 metric tons per year [2,3]. Additionally, a recreational fishery is conducted under an annual quota of 100 metric tons [4].

Red king crabs are recognized as hosts for a range of attached and mobile species, as is the case with several other crustacean groups [5,6,7]. The scientific literature describes a facultative association, identified as “epibiosis,” which takes place between two organisms, namely, the epibiont and the basibiont (or host). The term “epibiont” refers to organisms that are attached to the surface of a living substratum during the sessile phase of their life cycle. Meanwhile, the basibiont serves as the support for the epibiont [8]. In contrast, a “symbiont” is more closely connected with its host.

The study of epibiosis can enhance our understanding of key aspects of the biology of both hosts and associated species. The latter, which seldom have a free-living life stage and are challenging to collect using conventional methods, can benefit greatly from epibiosis research [9,10]. Moreover, analyzing fouling communities can yield valuable insights into the impact of epibionts on host populations [11,12,13,14]. Symbiotic and epibiotic interactions of the red king crab in its new habitat were studied at deep sites [15,16,17] and shallow-water locations [9,10,18,19]. Nevertheless, the majority of these investigations have been limited to the summer season. The comparison of red king crab infestation indices during different seasons may provide valuable information about the spatial variability of fouling communities and help to estimate the role of different factors in determining infestation rates. For this reason, the aims of the present study were (1) to describe the fouling community of red king crab in Sayda Bay, Russia, in winter, (2) to study some aspects of the biology of common associated species, and (3) to reveal seasonal changes in fouling communities of *P. camtschaticus* at the study site.

## 2. Materials and Methods

### 2.1. Study Area

**Figure 1:**
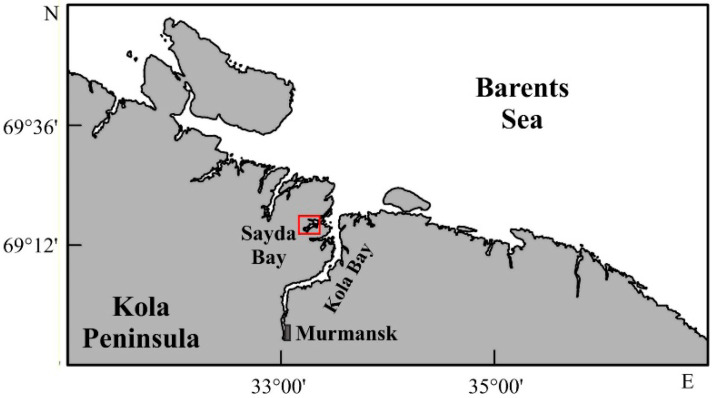
Location of Sayda Bay (red square), Russia, Barents Sea.

The study was carried out in Sayda Bay (69.2658° N, 33.2916° E), a fjord situated in the northwestern part of Kola Bay (Figure 1).

There is a deep-water sill in front of the study area with a minimum depth of 104 m. The maximum depth in Sayda Bay is 190 m. The lowest surface water temperature of 1 °C was recorded in March, while the highest temperature of 10 °C was observed in June. Salinity ranges between 32 psu in summer and 34 psu in winter. The initial ice coverage in Sayda Bay happens in November, with approximately 30% of the bay being covered in ice during the maximum ice extent in April. Ice-free waters are noted from mid-May to September [20].

This study area was chosen because we have a comparable dataset on the epibionts of red king crabs from Sayda Bay in the early summer period (late May–early June 2004) and in the autumn period (September 2005). These data have been partially presented in our previous paper [21].

### 2.2. Field Sampling and Data Analysis

A total of 97 crabs were collected on 12 November 2015 and 6 November 2016 by baited bottom traps at a 70 m depth. The conical traps with a mesh size of 25 mm baited with herring were set at the same locations as in our previous studies in 2004 and 2005 [21]. In the laboratory, each crab was sexed, and the carapace length (CL, the straight-line distance across the carapace from the posterior margin of the right eye orbit to the medial-posterior margin of the carapace) was measured. In addition, the molting stage was determined after Donaldson and Byersdorfer [22].

Associated organisms were removed from the crabs and preserved in 4% buffered formalin after their positions on the crab (i.e., on the carapace, limbs, abdomen, or mouth parts) were recorded. In the laboratory, associated organisms were counted (solitary species only) and identified. Morphometric analyses of barnacles (Cirripedia) included measurement of their basal diameters (BD, mm). Amphipods were sexed and their body lengths (BL, mm) were measured using an MBS-10 stereomicroscope (LOMO, Saint Petersburg, Russia) as per Dvoretsky and Dvoretsky [23].

To quantify the infestation of crabs, the following standard indices were used: (1) prevalence = proportion (%) of infested crabs; (2) intensity = mean number of fouling specimens per infested crab [24].

### 2.3. Statistical Analysis

Statistical data analysis was done using the STATISTICA 6.0 software package. Variations in percentage data (fouling prevalence and size-frequency distributions) were studied using chi-square tests. Non-parametric Kruskal–Wallis tests were applied to reveal differences between metric parameters (mean body lengths and infestation intensities) because normality and homoscedasticity in the data were not detected in most cases.

## 3. Results

**Figure 2:**
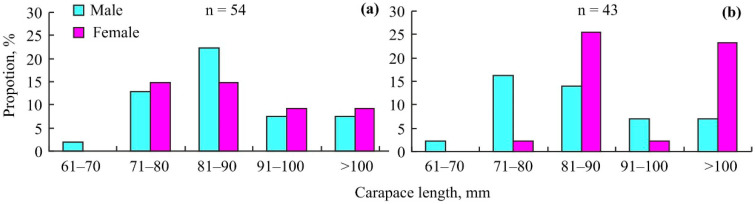
Size frequency distribution of the red king crab *Paralithodes camtschaticus* captured at a 70 m depth in Sayda Bay, Russia, in November 2015 (**a**) and 2016 (**b**).

Size-frequency distributions of the red king crabs that were caught in Sayda Bay are presented in Figure 2.

These distributions in November 2015 and 2016 were similar in males (df = 4, *p* = 0.913) and significantly different in females (df = 3, *p* = 0.018). In winter 2015–2016, mean red king crab CL (±SE) was 90.6 ± 1.6 (range 67.8–132.0) mm. This CL was significantly larger than the levels observed in autumn 2004 (85.4 ± 0.9, range 59.8–168.9) and in summer 2005 (84.4 ± 1.1, range 52.7–166.1) (df = 1, *p* < 0.001 in both cases). In November 2015 and 2016, all crab shells were determined to be “new shell” individuals (2–10 months post-ecdysis), which was in contrast to the crabs collected in September and May–June (2004–2005) when we registered the presence of males with “old shells” (>12 months post ecdysis).

A total of 12 associated species were observed on the crabs (Table 1).

**Figure 3:**
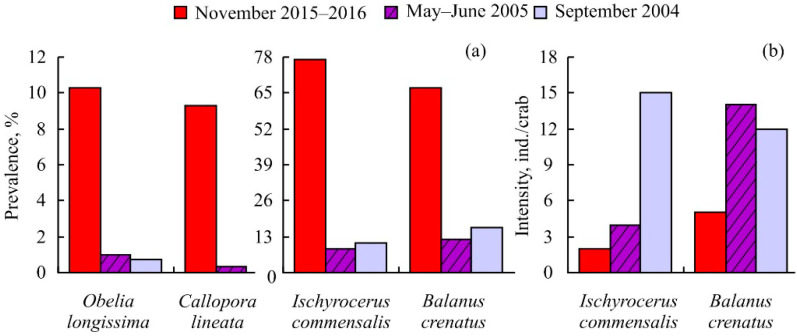
Infestation indices of common associated species on red king crabs *Paralithodes camtschaticus* captured in Sayda Bay, Russia, at different seasons. (**a**) The prevalence of infestation, (**b**) the median intensity of infestation.

Infestation indices of the common associates were similar in male and female crabs and did not differ significantly in 2015 and 2016 (df = 1, *p* > 0.05 in all cases). The most common associated organisms were the amphipod *Ischyrocerus commensalis* and the barnacle *Balanus crenatus.* The prevalences of common associated species were significantly higher in the winters of 2015 and 2016 than in the summer of 2005 and the autumn of 2004 (Figure 3a).

An opposite pattern was found for infestation intensities of the barnacles and amphipods (Figure 3b).

**Figure 4:**
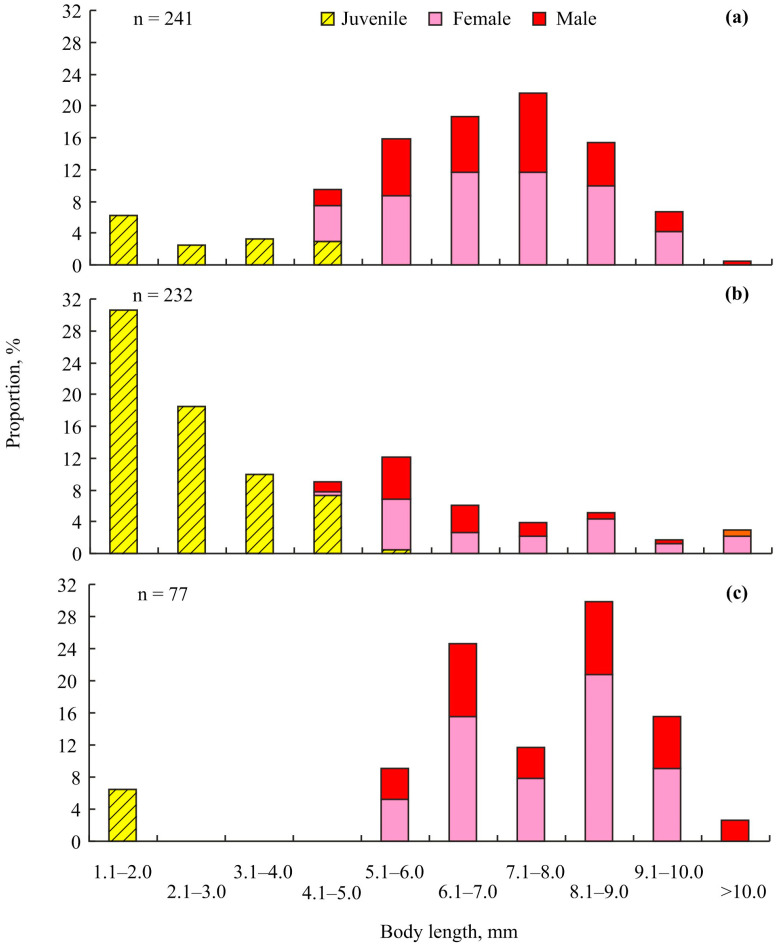
Size frequency distribution of amphipods *Ischyrocerus commensalis* that were found on red king crabs *Paralithodes camtschaticus* in Sayda Bay, Russia, in November 2015–2016 (**a**), September 2004 (**b**), and May–June 2004 (**c**).

The size-frequency distributions of *Ischyrocerus commensalis* during each season are shown in Figure 4.

These distributions differ significantly from each other (df = 9, *p* < 0.001 in all cases). The mean sizes of the amphipods were significantly different among all the considered seasons (df = 2, *p* < 0.001): the largest BL was found in May–June (7.4 ± 0.2 mm, range 1.4–10.5 mm), the medium BL in November (6.4 ± 0.1 mm, range 1.1–11.4 mm), and the smallest BL in September (4.0 ± 0.2 mm, range 0.8–11.2 mm).

**Figure 5:**
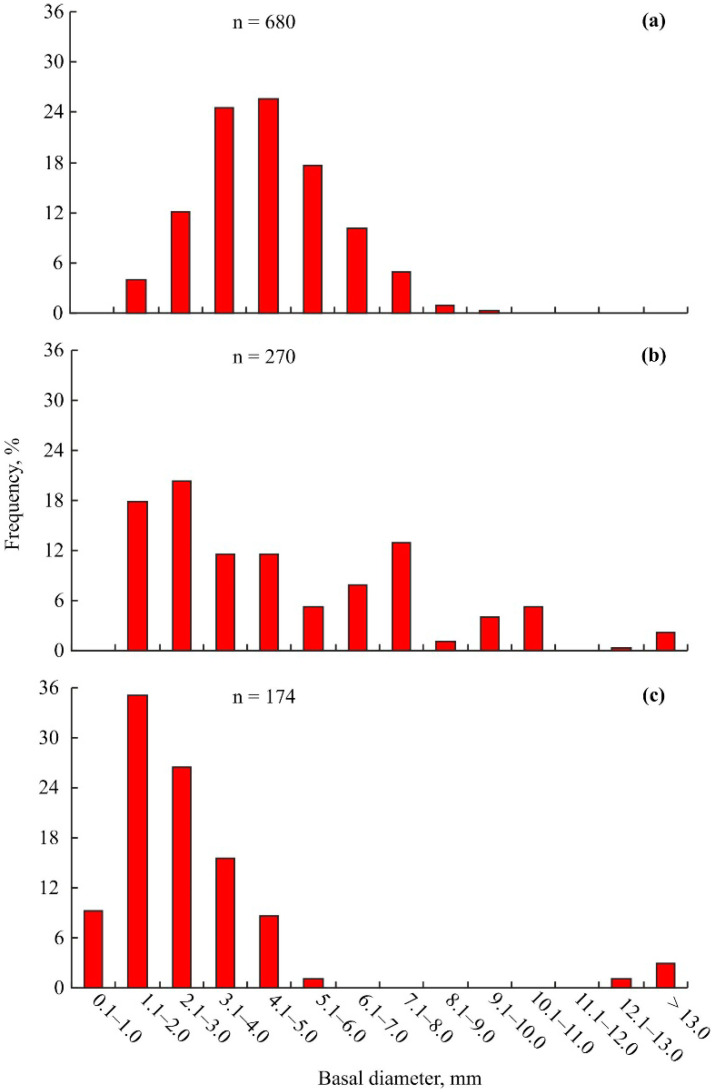
Size frequency distribution of barnacles *Balanus crenatus* that were found on red king crabs *Paralithodes camtschaticus* in Sayda Bay, Russia, in November 2015–2016 (**a**), September 2004 (**b**), and May–June 2004 (**c**).

Seasonal changes in the size-frequency distributions of *B. crenatus* individuals in Sayda Bay are presented in Figure 5.

Mean BD of *B. crenatus* in November (4.6 ± 0.1 mm, range 1.4–9.9 mm) was significantly higher than in May–June (2.8 ± 0.2 mm, range 1.0–15.0 mm) (df = 1, *p* < 0.001) and was similar to the September value (5.0 ± 0.2 mm, range 1.3–16.0 mm) (df = 1, *p* = 0.495).

The barnacle *B. crenatus* was most abundant on the carapace surface (66.6%) and the limbs (29.1%) of the crabs. The greatest percentage of the amphipod *I. commensalis* was found on the crabs’ mouth parts (90.8%). The hydrozoan *Obelia longissima* was most abundant on crab limbs (58.3%). Bryozoans dominated on the carapaces (80.0%). Similar preferences for crab carapaces were registered for copepods (63.6%) and spirorbids (66.7%).

## 4. Discussion

According to the size-at-age data of *Paralithodes camtschaticus* in the Barents Sea, the majority of crabs examined in the current study were 4–8-year-old specimens. The absence of crabs at age 0–2 years is explained by the limitations of our sampling gear, whose mesh size did not yield crabs smaller than 50 mm CL, and by the depth preferences of such small crabs relative to our study depths [25]. The lower proportions of large crabs (age 9–15 years) in November 2015–2016, compared to September 2004 and May–June 2005, are associated with migration patterns: after spawning (in May–April), mature males (CL > 100 mm) migrate to deep-water sites, and a similar but less pronounced pattern is registered for large females, which move to the open sea in late summer [1,26].

The majority of attached species observed on the crabs may be considered as epibionts. The predominance of barnacles on the crabs is not surprising because *B. crenatus* is one of the most common species in the coastal waters of the Kola Peninsula, where it usually settles on a rocky substrate at 2–200 m depths [27]. In the Barents Sea, the species has also been found on spider crabs (Oregonidae [27]) and northern stone crabs (*Lithodes maja* [19]). In other regions, barnacles in the genus *Balanus* are registered in fouling communities of hermit crabs (Paguroidea [12]), cancrid crabs (*Cancer* spp., [28]), snow crabs (*Chionoecetes opilio*; [29]), and shrimps (Caridea [30]).

The amphipod *I. commensalis* is a well-known symbiont of *P. camtschaticus* both in the Barents Sea [16,19,23] and in its native areas, e.g., in Alaska and near Sakhalin Island [31,32]. Other symbiotic species (the copepod *Tisbe furcata* and the hydrozoan *Coryne hincksii*) were also recorded on red king crabs in the coastal Barents Sea during our earlier surveys [19].

In Sayda Bay, the majority of barnacles and other attached species were observed on the crabs’ carapaces. This host site is the largest and smoothest area for colonization on the host, making it favorable for settlement by planktonic larvae [19]. In contrast, the predominance of *I. commensalis* on their hosts’ mouth parts is not surprising because these amphipods are mobile and feed on particles released or produced by their hosts: e.g., detritus, mucus, epibionts, and flesh [9,23].

We found that the winter prevalences of common associated species were higher than the summer and autumn levels (Figure 3a). This result could be explained by the fact that the proportion of small crabs (with zero infestation prevalences) in winter was lower than in other seasons. Similar patterns have been registered for different crab species such as *Arenaeus cribrarius* and *Cancer* spp. [28,33]. In contrast to prevalences of infestation, the mean intensities of barnacles and amphipods were lower in November than in September and May–June. This result could be associated with less favorable temperature conditions in winter than during spring and autumn. Temperature is known as a key factor determining the natural mortality of marine invertebrates [34,35,36,37].

Localization patterns of commonly associated species appear to mirror either their relationships with the host or larval settlement patterns. It is worth noting that *I. commensalis* feed on food remnants of red king crabs [23], and as such, most of these amphipods were observed on the mouthparts. By contrast, planktonic larvae of sessile benthic organisms do not establish close associations with potential hosts. Instead, these larvae were found to colonize the most accessible part of the host’s body, the carapace, which offers ample surface area and ease of settlement. Similar localization patterns have been observed in fouling communities of various crustaceans, e.g., *Hyas araneus*, *Cancer gracilis*, *Cancer productus*, and *Cancer magister* [19,28].

Winter data for *I. commenslis* expands our knowledge of the life cycle of this species [23]. In Sayda Bay, the majority of juvenile amphipods (size classes 1.1–2.0, 2.1–3.0, and 3.1–4.0 mm) were found in September (Figure 4b) after the peak of breeding in August. In November, the frequency distribution shifted so as to be dominated by the 5.1–6.0, 6.1–7.0, and 7.1–8.0 mm size classes (Figure 4a), indicating an approximately 2 mm BL growth rate per month. In contrast to August and May–June, we found only a few large *I. commensalis* individuals in November. This result can likely be attributed to the higher mortality of young and old amphipods owing to cold water temperatures during this period.

We found that, in November, the largest proportions of *Balanus crenatus* were registered in the 3.1–4.0 mm and 4.1–5.0 mm size classes (Figure 5a), while in September and May–June, the barnacles predominated in the 1.1–2.0 mm and 2.1–3.0 mm size classes (Figure 5b,c). The largest barnacles had a 9.9 mm BD. Settlement of *B. crenatus* occurs in April (when the majority of crabs have new shells) and in August. According to this information, the month growth increment in *B. crenatus* could be roughly estimated as 1.3 mm, i.e., the age of barnacles with a 9.1–10 mm BL was approximately 7 months. Size-at-age data obtained for *B. crenatus* could be useful in solving the problem of age determination of skipmolted male red king crabs (CL > 110 mm). Measuring the growth rate of epibiont barnacles to calculate or infer the intermolt duration of decapods is a well-known approach applied for host age determination worldwide [38,39,40].

## 5. Conclusions

In our survey conducted during late winter in Sayda Bay, we identified a total of 12 associated species that had colonized red king crabs. The dominant species among these were amphipods and barnacles, which exhibited higher prevalences compared to the summer months. This disparity can be attributed to seasonal fluctuations in the size-frequency distribution of red king crabs. With regard to other epibiotic organisms, infestation levels were found to be lower in winter, most likely due to less favorable temperature conditions. We quantified the monthly growth rates of *Balanus crenatus* barnacles and *Ischyrocerus commensalis* amphipods for the first time, obtaining measurements of 1.35 mm in basal diameter and 2 mm in body length, respectively. This newly acquired data contributes to our understanding of the biology of both the host species and its common symbionts. Furthermore, it has the potential to significantly impact the process of aging red king crabs in the Barents Sea.

## Figures and Tables

**Table 1:** Prevalence and mean intensity of fouling species found on red king crabs *Paralithodes camtschaticus* in Sayda Bay, Russia, in November 2015 and 2016.

Taxa	Prevalence, %	Mean Intensity, ind. per Crab		
		Mean ± SE	Min	Max
Hydrozoa				
*Coryne hincksii* Bonnevie, 1898	1.03	–	–	–
*Obelia longissima* (Pallas, 1766)	10.31	–	–	–
Nemertini				
*Nemertini* g. sp.1	1.03	1.0 ± 0.0	1	1
Polychaeta				
*Circeis armoricana* Saint-Joseph, 1894	4.12	1.5 ± 0.3	1	2
Bivalvia				
*Mytilus edulis* L., 1758	3.09	1.7 ± 0.3	1	2
Copepoda				
*Ectinosoma normani* Scott T. & A., 1894	3.09	1.0 ± 0.0	1	1
*Mesochra pygmaea* (Claus, 1863)	4.12	1.0 ± 0.0	1	1
*Tisbe furcata* (Baird, 1837)	4.12	1.0 ± 0.0	1	1
Amphipoda				
*Ischyrocerus commensalis* Chevreux, 1900	77.32	1.7 ± 0.1	1	4
Cirripedia				
*Balanus crenatus* Brugiere, 1789	67.01	14.3 ± 2.7	1	94
Bryozoa				
*Callopora lineata* (L., 1767)	9.28	–	–	–
*Scrupocellaria arctica* (Smitt, 1868)	1.03	–	–	–

## Data Availability

The data presented in this study are available on request from the corresponding author. The data are not publicly available due to privacy restrictions.
